# Amplifications of Stemness Gene Loci—New Markers for the Determination of the Need for Neoadjuvant Chemotherapy for Patients with Breast Cancer. A Prospective Study

**DOI:** 10.3390/jpm11050397

**Published:** 2021-05-11

**Authors:** Nikolai V. Litviakov, Marina K. Ibragimova, Matvey M. Tsyganov, Polina V. Kazantseva, Artem V. Doroshenko, Eugeniy Yu. Garbukov, Irina G. Frolova, Elena M. Slonimskaya

**Affiliations:** Cancer Research Institute, Tomsk National Research Medical Center of the Russian Academy of Sciences, 634050 Tomsk, Russia; imk1805@yandex.ru (M.K.I.); tsyganovmm@yandex.ru (M.M.T.); polydoctor@yandex.ru (P.V.K.); doroshenko_artem@icloud.com (A.V.D.); jrmaximum@rambler.ru (E.Y.G.); frolovaig@oncology.tomsk.ru (I.G.F.); slonimskaya@rambler.ru (E.M.S.)

**Keywords:** breast cancer, neoadjuvant chemotherapy, stemness genes, markers of metastasis, prospective study

## Abstract

In this prospective study, a new strategy for the prescription of neoadjuvant chemotherapy (NAC) was prospectively tested and depended on the presence of stemness gene amplifications in the tumor before treatment, which in our early studies showed a connection with metastasis. The study included 92 patients with grade IIA–IIIB luminal B breast cancer. Patients underwent a biopsy before treatment, and with the use of a CytoScan HD Array microarray (Affymetrix, Santa Clara, CA, USA), the presence of stemness gene amplifications (3q, 5p, 6p, 7q, 8q, 13q, 9p, 9q, 10p, 10q21.1, 16p, 18chr, 19p) in the tumor was determined. In group 1 (*n* = 41), in the presence of two or more amplifications, patients were prescribed a personalized NAC regimen. In group 2 (*n* = 21), if there was no amplification of stemness genes in the tumor, then patients were not prescribed NAC, and treatment began with surgery. Group 3 (*n* = 30) served as a historical control. The frequency of an objective response to NAC in groups 1 and 3 was 79%. Nonmetastatic survival was found in 100% of patients in group 2, who did not undergo NAC. In patients in group 1, the frequency of metastasis was 10% (4/41). At the same time, in patients in group 3, who received NAC, the rate of metastasis was 47% (14/30). The differences between group 1 and group 3 and between group 2 and group 3 were statistically significant, both by Fisher’s criterion and a log-rank test. The appointment of NAC was most feasible in patients with clones with stemness gene amplifications in the primary tumor, while in the absence of amplifications, preoperative chemotherapy led to a sharp decrease in metastasis-free survival. This strategy of NAC prescription allowed us to achieve 93% metastatic survival in patients with breast cancer.

## 1. Introduction

Currently, an increasing number of works are devoted to the fact that chemotherapy and targeted therapy in some patients can stimulate the formation of new mutations, which leads to the formation of treatment resistance and tumor metastasis [1,2]. Such data were presented for many types of cancer, such as acute lymphoblastic leukemia [3], chronic lymphocytic leukemia [4], acute myeloid leukemia [5], esophageal adenocarcinoma [6], glioblastoma [7], and lung cancer [8]. Neoadjuvant chemotherapy in some patients stimulated breast cancer metastasis [9] and facilitated the progression of the metastatic process: stimulating EMT (epithelial-mesenchymal transition) in tumor cells, invasion, intravasation and inflammation [10]. It has also been demonstrated in experimental models that, in some cases, chemotherapy can significantly enhance the invasive properties of tumor cells and promote the formation of metastatic niches and the ability to extravasate [11,12]. NAC elimination of mutated tumor cells without the enhancement of mutagenesis leads to a favorable outcome. The breast tumors or 364 patients were studied before treatment and after neoadjuvant chemotherapy. The frequency of somatic mutations in *TP53* or *PIK3CA* was halved during NAC (from 24.8% before treatment to 12.1% after NAC, *p* < 0.001). If patients with *TP53* or *PIK3CA* mutations in the tumor before treatment became negative for these mutations after chemotherapy, they showed significantly better overall and disease-free survival (*p* = 0.008) [13]. Thus, preoperative chemotherapy is a kind of competition between the rate of the elimination of tumor cells and the rate of clonal chemoinduced evolution of tumor cells.

**Figure 1:**
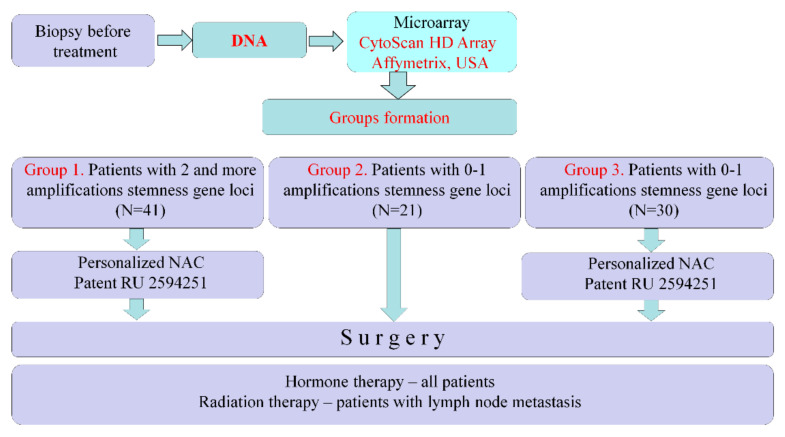
Prospective study design.

Our studies have shown that various CNAs (copy number aberrations), deletions and/or amplifications can occur in a breast tumor during NAC. At the same time, the emergence of amplifications of the regions of localization of stemness genes (3q (26.33), 5p (15.33; 13.1), 6p (24.3; 22.3; 21.33; 21.32), 7q (11.23; 21.13; 31.2; 32.1), 8q (11.21; 24), 9p (21.2), 9q (34.3; 21.13; 31.2, 22.33), 10p (15.2; 13; 12.2; 11.22), 10q22.1, 12p (13.31) 13q (34; 32.3; 22.1; 13.3; 12.2), 16p (11.2; 13.3), 18q (21.1; 21.2) 19p (13.3; 13.2; 13.12) by treatment NAC or the remainder of two or more amplifications in a post-NAC tumor, regardless of the initial level, is tied with a high incidence of metastasis (92% and 52%, respectively). In the absence of amplifications or with only one amplification in the tumor after NAC, not a single patient had metastases [14]. The elimination of tumor clones with stemness gene amplification using NAC also leads to 100% metastasis-free survival [15]. The presence of amplifications of stemness gene loci in a tumor before treatment also has prognostic significance. NAC in patients with breast cancer who do not have stemness gene amplifications in the primary tumor or who have one amplification, regardless of the effectiveness of NAC, worsens metastatic survival, which makes NAC inappropriate for such patients. Metastatic-free survival in patients with two or more amplifications of stemness genes in a tumor before treatment depends on the effect of NAC, and with complete and partial regression, metastatic survival is significantly higher than with stabilization and progression [16]. In this work, a prospective study, a new strategy for the prescription of NAC was tested; NAC was prescribed only to patients who had two or more stemness gene amplifications in their tumors. If there were no stemness gene amplifications in the tumor before treatment or if there was only one amplification, then the treatment of such patients began with surgery. The prospective study design is shown in Figure 1.

Patients underwent biopsy before treatment and had their DNA isolated. We studied DNA using a CytoScan HD Array (Affymetrix, USA) and determined the presence of stemness genes in tumors. In cases of amplifications at two or more chromosomal regions of stemness gene localization (in different chromosomes), patients were assigned personalized NAC (Patent RU 2594251) to eliminate tumor clones with stemness genes amplifications. Such patients were assigned to Group 1 (*n* = 41). If, according to the results of the microarray study, tumors manifested no stemness gene amplifications or amplification at only one chromosome region, then such patients were not prescribed NAC and had their treatment began with surgery. These patients were assigned to Group 2 (*n* = 21). Group 3 (historical control) consisted of 30 patients with luminal B HER2-negative breast cancer who had no stemness gene amplifications or had an amplification of only one chromosome region in their tumors prior to treatment. Patients underwent personalized NAC according to Patent RU 2594251, followed by surgery and standard postoperative care in all groups. The metastasis-free survival rate was estimated. (Figure 1).

## 2. Result

To show the significance of stemness gene amplifications for tumor metastasis, this prospective study involved 92 breast cancer patients. In the prospective study, three groups were formed, which did not differ in their main clinical and morphological indicators (tumor extent (T and N), molecular subtype) but differed in treatment approaches (Table 1). Groups 1 and 3 underwent personalized NAC according to similar schemes, while Group 2 did not. Groups 2 and 3 differed from group 1 by the fact that these patients did not manifest stemness gene amplifications in their tumors before treatment or there was only one amplification, while patients in group 1 had two or more stemness gene amplifications in their tumors.

As a result of the personalized NAC in groups 1 and 3, the same frequency of objective response to NAC was achieved by the sum of full and partial regressions (Table 1). It should be noted that the frequency of an objective response to NAC in groups 1 and 3 with a personalized approach to prescribing a treatment regimen was 79% in both groups.

**Figure 2:**
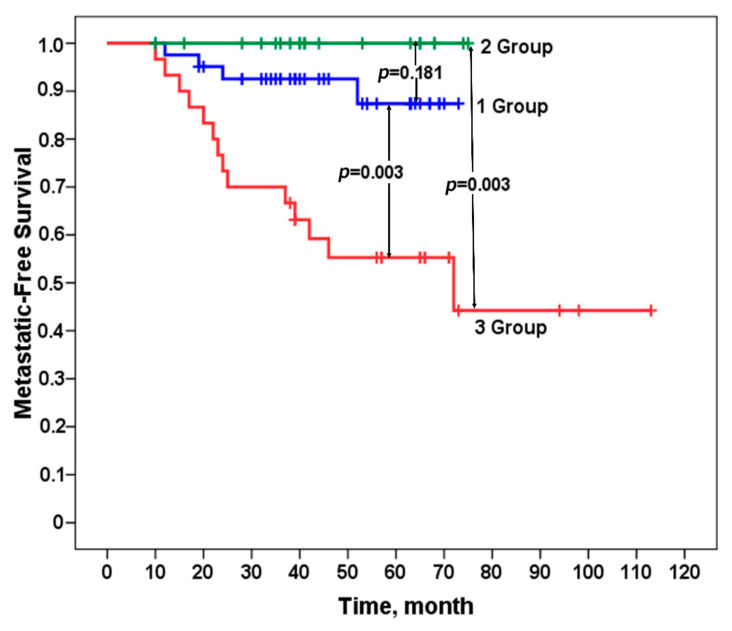
Metastasis-free survival in patients in groups 1–3 in the prospective study. *p*-value—log-rank test.

We evaluated the metastasis-free survival rate of patients in the studied groups according to the Kaplan–Meier method. A 100% metastatic survival rate was established in patients in group 2 who did not have stemness gene amplifications in their tumors or had only one amplification and did not undergo NAC. For patients in group 1 with two or more amplifications at regions of stemness gene localization who received personalized NAC, the incidence of metastasis was not high, amounting to 10% (4/41). Patients in group 3, who had no stemness gene amplifications in their tumors or had only one amplification, underwent personalized NAC, which led to an incidence of metastasis as high as 47% (14/30). The differences between group 1 and group 3 were statistically significant, as well as between group 2 and group 3, both by Fisher’s criterion (Table 1) and a log-rank test (Table 1 and Figure 2). The *p*-value for all three log-rank (Mantel–Cox) groups is 0.00026.

At this stage, we performed a sample study of residual tumors in patients in groups 1 and 3 and established that in 65% of patients, personalized NAC eliminated the originally existing clones with stemness gene amplifications in patients in group 1; at the same time, it contributed to the emergence of new clones with stemness gene amplifications in patients in group 3 in 40% of cases.

**Figure 3:**
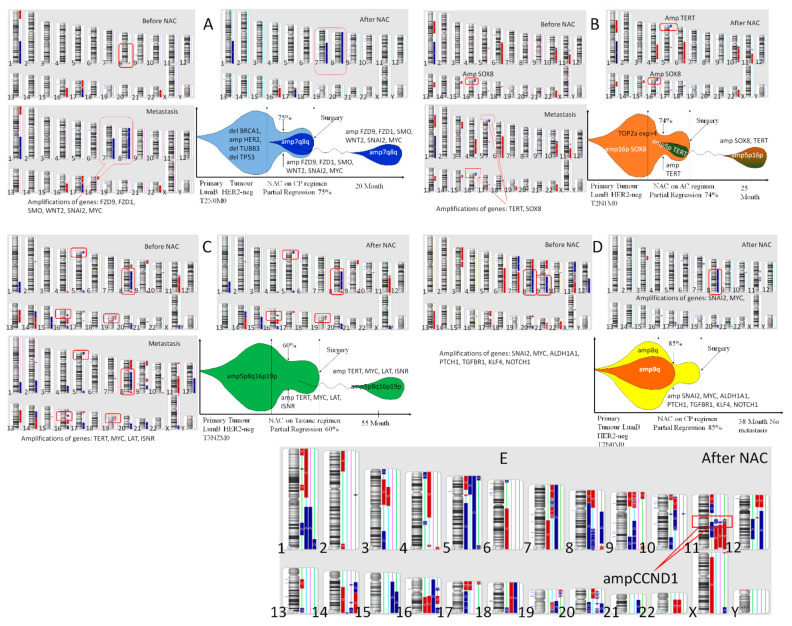
CNA genetic landscape of the tumor before treatment and after NAC, and metastasis. (**A**) The K1 patient of Group 3, 47 years of age, with luminal B left breast cancer T_2_N_0_M_0_, underwent four courses of CP treatment with 75% partial regression, surgery without complications, hormonal therapy after surgery; a metastatic tumor was found in the liver 20 months later. (**B**) The B2 patient of Group 3, 50 years of age, with luminal B right breast cancer T_2_N_1_M_0_, underwent six courses of AC treatment with 74% partial regression, surgery without complications, hormonal therapy and radiotherapy after surgery; a metastatic tumor was found in the lung 25 months later. (**C**) The Ch1 patient of Group 1, 32 years of age, with luminal B right breast cancer T_3_N_2_M_0_, underwent six courses of taxane treatment with 60% partial regression, surgery without complications, hormonal therapy and radiotherapy after surgery; a metastatic tumor was found in the liver 55 months later. (**D**) The S2 patient of Group 1, 45 years of age, with luminal B right breast cancer T_2_N_0_M_0_, underwent six courses of CP treatment with 85% partial regression, surgery without complications, hormonal therapy after surgery; a metastatic tumor was not found 38 months later. (**E**) The CNA genetic landscape of the tumor after NAC of four patients of Group 1 with metastasis.

One of the cases of the appearance of clones with amplifications of stemness genes in the process of NAC is presented in Figure 3, which shows the CNA genetic landscape of the primary tumor before treatment and after NAC, hematogenic metastasis, and the model of clonal evolution of the K1, B2, Ch2, S2 patients (A–D) and the CNA genetic landscape of the residual tumor after NAC of 4 patients of Group 1 with metastasis. The K1 patient of Group 3 had no stemness gene amplification before treatment, and NAC, despite partial regression of 75%, induced the formation of 7q and 8q amplifications; the same amplifications were found in the liver metastasis (Figure 3A). The B2 patient of Group 3 had 1 stemness gene amplification *SOX8* (16p) before treatment, and NAC, despite partial regression of 74%, induced the formation of amplification of stemness gene *TERT* in 5p locus; the *TERT* and *SOX8* amplifications were found in the lung metastasis (Figure 3B).

The Ch1 patient of Group 1 had four stemness genes amplifications before treatment (*TERT*-5p, *MYC*-8q, *LAT*-16p and *INSR*-19p), and NAC, despite partial regression of 60%, could not eliminate of none of the above amplifications; the same amplifications were of 55 months after treatment und in the liver metastasis (Figure 3C). Against, the S2 patient of Group 1 had 7 stemness genes amplifications (*SNAI2*-8q, *MYC*-8q, *ALDH1A1*-9q, *PTCH1*-9q, *TGFBR1*-9q, *KLF4*-9q, *NOTCH1*-9q) before treatment, and NAC, at partial regression of 86%, destroyed of amplification 9q (*ALDH1A1*-9q, *PTCH1*-9q, *TGFBR1*-9q, *KLF4*-9q, *NOTCH1*-9q) (Figure 3D). This has provided metastasis-free survival for 38 months for the patient S2 and the patient is further observed. Figure 3E shows the CNA genetic landscape of the residual tumor of four patients in group 1 who developed metastases. In addition to the presence of two or more amplifications of the stemness genes in each tumor, all patients had an amplification of the *CCND1* gene. However, this amplification of the *CCND1* gene were also observed in some other patients in this group who did not metastasize. These data confirm the importance of stemness gene amplifications for the metastatic process.

## 3. Discussion

Apparently, it is time to review the indications for NAC and to emphasize other aspects of treatment, which would take the rate of chemoinduced evolution of tumor cells in the patient and the probability of the formation of treatment resistance and tumor metastasis into account. The appointment of the treatment should be personalized, and it is necessary to predict the stimulation of metastasis in the patient. We managed to identify markers of metastasis prediction—they are focal amplifications at regions of stemness gene localizations (3q, 5p, 6p, 7q, 8q, 13q, 9p, 9q, 10p,10q21.1, 16p, 18chr, 19p), and the initial results of our prospective studies showed the success of their clinical application, at least in terms of the appointment of NAC to breast cancer patients. Due to prospective research, we obtained the most significant evidence for the predictive significance of stemness gene amplifications for metastasis. The abolition of preoperative chemotherapy for patients without stemness gene amplifications in tumors significantly improved their survival rate, and the elimination of clones with stemness gene amplifications by means of NAC led to metastatic disease that did not develop. Those amplifications of these chromosomal loci are associated with unfavorable outcomes [17,18,19,20].

In Figure 3, we show clinical cases of clones with stemness gene amplifications in tumors after NAC, and the same amplifications were observed in a metastatic tumor that developed 28 months later. Similar data were put forth by Ng, C.K., et al. (2017), who presented nine cases of CNA genetic landscape study of primary tumors and their metastases in Figure 3. Significant heterogeneity was observed between the primary tumors and the metastases, but all metastases had two or more amplifications at regions of stemness gene localization, which are found in the primary tumor [21]. This finding allows us to assume that all tumors had the ability to metastasize due to amplifications, and it was the clones with amplifications that gave rise to the process of metastasis.

We believe that CNA testing of the genetic landscape of breast tumors for NAC prescription can be performed routinely in all operable luminal B breast cancer patients, and it can be assumed that it can be used for other types of breast cancer, but this requires further research.

## 4. Materials and Methods

### 4.1. Patients

A total of 92 breast cancer patients were included in the prospective study, which began in 2014. Inclusion criteria: newly diagnosed/untreated patients were enrolled, grade IIA–IIIB (*T*1–3*N*0–2*M*0); luminal B HER2-negative breast cancer; age 32 to 63 years (average age 45.3 ± 0.6); without *BRCA1* and/or *BRCA2* germinal mutations. Exclusion criteria: grade IA-IB or IIIA–IV, luminal A, luminal B HER2-positive, TNBC or HER2-positive molecular subtype of breast cancer; ageless 30 years or more 65 years; presence *BRCA1* and/or *BRCA2* germinal mutations.

The procedures followed in this study were performed in accordance with the Declaration of Helsinki (1964, amended in 1975 and 1983). This study was approved by the institutional review board, and all patients signed an informed consent form for voluntary participation.

The molecular subtype of before-NAC breast tumor was defined immunohistochemistry. Luminal B subtype of breast cancer was showed expression of ER and PR. The Ki67 expression were more 30% and HER2 expression was absent. To determine the clinical response of the tumor to NAC, the volume of the primary tumor was assessed using ultrasound and mammography before treatment, after 2 courses of NAC and after the end of chemotherapy. The clinical tumor response was classified according to the International Cancer Union criteria: clinical complete response (CR), partial response (PR), stable disease (SD), progressive disease (PD). Surgical material of the tumor after NAC was examined morphologically and complete pathological response (pCR) was determined using the RCB criteria (RCB-0). After surgery, in the amount of mastectomy or resection (80% of cases), patients received standard therapy. All patients received hormonal therapy, in addition, patients with lymph node metastases received standard radiation therapy.

### 4.2. DNA Isolation

DNA was extracted from tumor tissues of pre- and post-NAC samples with the QIAamp DNA Mini Kit (Qiagen, Germany #51304).

### 4.3. Microarray Analysis

The CNA genetic landscape of tumor samples before and after NAC was outlined using Affymetrix microarrays (Santa Clara, CA, USA) CytoScanTM HD Array (http://www.affymetrix.com/esearch/search.Jsp?Pd=prod520004&N=4294967292) in accordance with protocol. The GeneChip^®^ Scanner 3000 7G (Affymetrix) was used to read the chips, the Chromosome Analysis Suite 4.1 software (Affymetrix) was used to analyze the microarray data and determined losses and gains of chromosomal loci.

### 4.4. RNA Isolation

Tumor biopsy before treatment and surgical material after NAC were preserved with RNA-Later solution (Sigma, St. Louis, MO, USA). RNA was isolated using the RNeasy Mini Kit Plus (Qiagen, Germany Cat. No./ID: 74034) according to the instructions. RNA quality was assessed using capillary electrophoresis on an instrument TapeStation (Agilent Technologies, Santa Clara, CA, USA).

### 4.5. Assessment of Gene Expression

*TOP2A*, *TYMS*, and *TUBB3* were evaluated with the previously described [22] quantitative real-time reverse transcriptase PCR (RT-qPCR) with TaqMan technology on a RotorGene-6000 amplifier (Corbett Research, Mortlake, NSW, Australia). To obtain cDNA on an RNA template, a reverse transcription reaction was performed with a Revert Aid ™ kit (Thermo Fisher, Waltham, MA, USA) with random hexanucleotide primers.

### 4.6. Personalized NAC Prescription Technique

**Figure 4:**
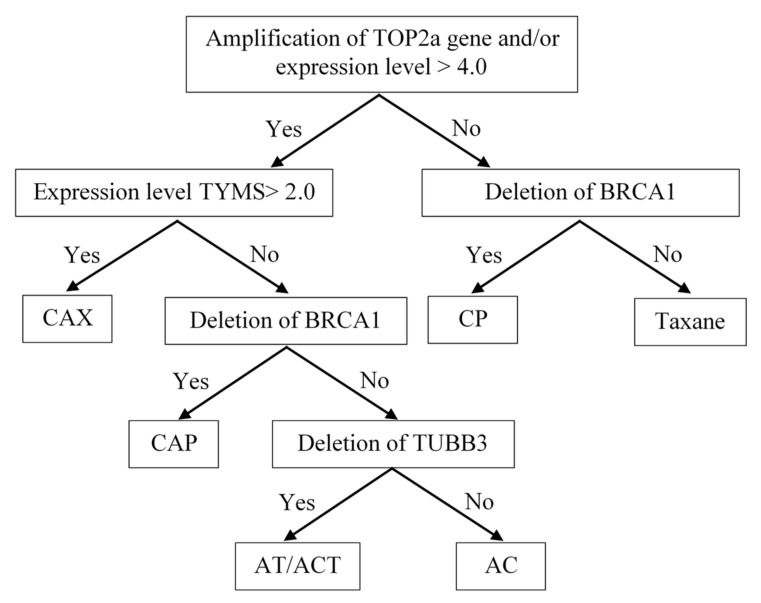
Algorithm for personalized prescription of NAC to patients with breast cancer. NAC regimen: CAX—cyclophosphamide, doxorubicin, Xeloda; CP—cyclophosphamide, cisplatin; CAP—cyclophosphamide, doxorubicin, cisplatin; AT—doxorubicin, docetaxel; ACT—doxorubicin, cyclophosphamide, docetaxel AC—doxorubicin, cyclophosphamide; Taxane—Taxotere in monotherapy.

For personalized NAC prescription, microarray analysis of tumor DNA was performed on a CytoScan HD Array chip (Affymetrix), and the expression level of the *TOP2a*, *TYMS*, and *TUBB3* genes was evaluated with qPCR. According to the copy number of the *TOP2a*, *TUBB3*, *BRCA1* genes and the level of expression of the *TOP2a*, *TYMS* and *TUBB3* genes in the tumor of each patient, the NAC scheme was personalized before treatment. The algorithm for personalized selection of an NAC scheme is presented in Figure 4 (Patent RU 2594251).

Indications for the use of anthracyclines were the presence of the amplification of the *TOP2a* gene locus in the tumor tissue, as well as its high expression level of more than 4.0. The question of the feasibility of capecitabine prescription arose when anthracycline drugs were indicated. The main criterion confirming the need for capecitabine was a high expression level of *TYMS* ≥ 2.0. The presence of a *BRCA1* gene deletion in the tumor tissue determined the indication for the prescription of regimens with the inclusion of platinum-based drugs and contraindications for prescribing taxanes. In determinations of the indications for prescribing taxanes, a low level of *TUBB3* expression and a deletion in the *TUBB3* gene locus (16q24.3) were used as predicting factors.

The personalized choice of NAC regimen had the following hierarchical organization: the leading role was played by the determination of indications for the prescription of anthracyclines, namely, the amplification of the *TOR2a* gene and/or its expression level ≥4.1. This determined the possibility of the use of regimens such as FAC, CAX, CAP, and AT/ACT. Then, the decision on the advisability of the use of capecitabine was made. The CAX regimen was prescribed when there was a high level of expression of the *TYMS* gene in the tumor tissue, and when the expression level was low, the state of the *BRCA1* gene locus became decisive in the choice of a regimen. With the deletion of this gene, platinum-based drugs were prescribed. In the case of indications for both anthracyclines and platinum, CAP regimens were prescribed. When *TUBB3* gene deletion was detected in patients for whom anthracyclines were recommended, the AT or ACT regimen was used. If there was evidence in the tumor tissue only for the prescription of anthracycline drugs, an AC regimen was recommended.

In the absence of indications for the appointment of anthracyclines, the state of the BRCA1 gene locus was decisive. Its deletion determined the appropriateness of the use of platinum-based drugs and was a contraindication for the use of taxanes—the CP regimen. In the absence of *TOP2a* amplification, low expression and a lack of *BRCA1* deletion, taxanes were prescribed in mono mode.

### 4.7. Statistical Analysis

A *p-*value was calculated with Fisher’s exact test (http://vassarstats.net/odds2x2.html, accessed on 10 November 2020, Free), and a one-sided *p*-value was calculated with the chi-squared test (http://vassarstats.net/, accessed on 10 November 2020, Free). Metastatic-free survival was calculated with the Kaplan–Meier method, and differences among patient groups were evaluated with a log-rank test. Statistical analyses were performed with SPSS Statistic 17.0.

## 5. Conclusions

Our retrospective [1,15] and prospective studies convincingly showed that if there were no stemness gene amplifications in breast tumors, the tumors did not metastasize and there was no need for preoperative chemotherapy, and the removal of the primary tumor site was sufficient; on the contrary, NAC could contribute to the stimulation of metastasis. In this regard, the appointment of NAC was most feasible in patients with clones with stemness gene amplifications in the primary tumor, while in the absence of amplifications, preoperative chemotherapy led to a sharp decrease in metastasis-free survival. This strategy of NAC prescription allowed us to achieve unexampled 93% metastasis-free survival in patients with breast cancer.

## Figures and Tables

**Table 1:** Comparison of clinical indicators of patients in prospective study groups.

Parameters		1 Group, *n* = 41	2 Group, *n* = 21	3 Group, *n* = 30	*p*-Value
Tumor size	T1	4 (10%)	1 (5%)	2 (7%)	# *p*1–3 = 0.901
	T2	33 (80%)	17 (81%)	25 (83%)	# *p*1–2 = 0.712
	T3	4 (10%)	3 (14%)	3 (10%)	# *p*2–3 = 0.869
Lymph node status	N0	21 (51%)	10 (48%)	12 (40%)	* *p*1–3 = 0.470
	N1-2	20 (49%)	11 (52%)	18 (60%)	* *p*1–2 = 1.0
					* *p*2–3 = 0.774
Molecular type	Lum B HER2-neg	41 (100%)	21 (100%)	30 (100%)	
NAC regimen	AC	8 (20%)	-	10 (33%)	# *p* = 0.329
	CAX	7 (17%)	-	6 (20%)	
	Taxotere	7 (17%)	-	5 (17%)	
	AT	10 (24%)	-	2 (7%)	
	CAP/CP	9 (22%)	-	7 (23%)	
NAC response	pCR	9 (22%)	-	5 (17%)	# *p* = 0.558
	PR	24 (59%)	-	19 (63%)	
	SD	6 (14%)	-	6 (20%)	
	PD	2 (5%)	-	0 (0%	
Frequency of Metastasis		4 (10%)	0 (0%)	14 (47%)	* *p*1–3 = 0.0007
					* *p*1–2 = 0.290
					* *p*2–3 = 0.0002
Median time patients were followed, month (M ± SE)		45.6 ± 2.7	44.9 ± 4.4	50.7 ± 5.4	† *p*1–3 = 0.365
					† *p*1–2 = 0.868
					† *p*2–3 = 0.435

Note: * *p*-value Fisher’s exact test (http://vassarstats.net/odds2x2.html, accessed on 10 November 2020, Free), # *p*-value chi-squared test (http://vassarstats.net/, accessed on 10 November 2020, Free), † *p*-value *t*-test independent, by variables. *p*1–3—*p*-value between 1 and 3 groups.

## Data Availability

Database registration certificate RU 2019620731 03/11/2019 Ibragimova, M.K., Tsyganov, M.M., Deryusheva, I.V., Kazantseva, P.V., Slonimskaya, E.M., Litvyakov, N.V. Database of changes in the amplification of stemness genes in breast tumors during preoperative therapy.
